# The Use of Allograft Bone in the Lateral Approach of Sinus Floor Elevation: A Systematic Review of Clinical Studies

**DOI:** 10.3390/medicina60020252

**Published:** 2024-01-31

**Authors:** Alexandra-Camelia Pogacian-Maier, Alexandru Mester, Rares-Luca Morariu, Radu Septimiu Campian, Andrei Tent

**Affiliations:** 1 Department of Oral Health, University of Medicine and Pharmacy “Iuliu Hatieganu”, 400012 Cluj-Napoca, Romania; maier.alexandra.camelia@elearn.umfcluj.ro (A.-C.P.-M.); rares.luca.morariu@elearn.umfcluj.ro (R.-L.M.); rcampian@email.com (R.S.C.); 2 Doctoral School, University of Medicine and Pharmacy “Iuliu Hatieganu”, 400347 Cluj-Napoca, Romania; 3 Department of Oral and Maxillofacial Surgery, University of Oradea, 410087 Oradea, Romania; tent_andrei@yahoo.com

**Keywords:** sinus lift, sinus floor elevation, allograft, bone regeneration

## Abstract

*Background and Objectives*: The aim of this systematic review was to assess the efficiency of using allografts for sinus lift. *Materials and Methods*: This systematic review was written under the Preferred Reporting Items for Systematic Reviews and Meta-Analyses (PRISMA) guidelines and recommendation of the Cochrane Handbook for Systematic Reviews of Interventions. Three electronic databases were screened until October 2023. The risk of bias was assessed according to the Strengthening the Reporting of Observational studies in Epidemiology (STROBE) guidelines. Statistical analysis was performed for median bone volume and implant survival rate. *Results*: From 321 articles retrieved, 7 articles were included in this review. A comparison between freeze-dried bone allograft (FDBA) and deproteinized bovine bone (DBB) for mean bone volume indicated a weighted mean difference (WMD) of −0.17 [−0.69, 0.36] (95% confidence interval (CI)), *p* = 0.53. For implant survival rate, a comparison was made between FDBA and autogenous bone indicating a risk ratio (RR) of 1.00 [0.96, 1.05] (95% CI), *p* = 1.00. *Conclusions*: The available evidence suggested that allograft bone can be used in sinus lift procedures. The results obtained are insufficient to compare with other types of bone graft, requiring a longer follow-up time. Future clinical trials are needed in order to evaluate the advantages of using allograft bone.

## 1. Introduction

The external sinus lift is considered a safe surgical method for bone augmentation in the molar area in order to gain vertical bone length for the insertion of dental implants [1,2,3]. The survival rate of dental implants in time is dependent on several factors that must act convergently: the chosen surgical technique and possible complications secondary to dental extractions prior to implantation, the time elapsed since tooth extraction, as well as the postoperative healing process [2]. Also, the osteoregenerative potential at this level should not be neglected either, which may differ from one individual to another and may be influenced by a series of associated pathologies [3]. The possibilities of performing sinus lift are multiple, depending on the surgical approach, the type of grafting materials, as well as the timing of dental implant insertion [1]. Depending on the bone supply and the chosen technique, the implants can be inserted postextraction, at the same time, or at 4 to 6 months after sinus floor bone augmentation at this level [3]. The choice of the bone-grafting material has been widely debated over time, with the authors’ opinions being often divided [1,2,3]. To ensure the safety of the surgical intervention, as well as to evaluate the anatomical characteristics of both the recipient and the donor sites, currently, an imaging examination of both anatomical sites is necessary [4]. Cone-beam computed tomography (CBCT) provides us with a good three-dimensional bone evaluation, being considered the gold standard in the planning of maxillary sinus surgery [4].

The approach to the maxillary sinus floor may be either transcrestal or through the lateral wall of the maxilla, with the second method being more invasive [5]. However, due to the fact that the elevation of the sinusal membrane is performed in sight, this method is considered safer [4,5]. It is also considered the method of choice for extensive posterior maxillary bone resorptions, where multiple bone grafts are required [4,5]. Numerous studies have been carried out in order to identify, at the level of the receptor site, which anatomical structure exhibits the greatest osteoinductive and osteoregenerative potential [4]. Following these studies, it was demonstrated that, in addition to the major osteoregenerative potential of the sinus walls, the sinus membrane also has similar properties [6]. The most common intraoral donor regions are the maxillary tuberosity, the chin region of the mandible as well as its external oblique line, and the vertical mandibular ramus [6]. 

For a long time, the use of autogenous bone grafts was considered the best choice due to their superior osteoinductive and osteoconductive properties [4]. Autografts can be harvested both from the oral environment and from neighboring extraoral donor areas [4]. From the extraoral regions, we mention the tibia, the calvaria, and most frequently the anterior iliac crest [4]. The anterior iliac crest ensures an extensive supply from which to harvest autologous bone, including cortical and cortico-medullary bone, being the donor site of choice for extended maxillary reconstructions [7]. The advantages of harvesting bone from this level is the reduced donor morbidity, as well as the fast postoperative recovery of the donor site, compared to other anatomical locations [6,7].

However, the disadvantage of morbidity at the level of the donor site cannot be neglected [5]. For these reasons, the use of biomaterials with bone-regenerating properties, such as tricalcium phosphate (TCP) or calcium sulfate (CS), has emerged. These materials are osteoinductive and are, over time, replaced by new bone under the conditions of ensuring a well-vascularized receptor bed [6]. Their association with platelet-rich fibrin (PRF) was tried, attaining successful outcomes in the majority of cases [6]. The PRF plays an important role in improving vascularization and bone remodeling, contributing substantially to increasing the effect of these biomaterials [6]. In severe posterior maxillary atrophies, these materials can present biological instability and accelerated resorption over time [7]. These shortcomings can lead, over time, to the impossibility of inserting dental implants if the vertical dimension is not sufficient, or even to their more rapid loss over time [7]. Also, different xenografts, such as deproteinized bovine bone (DBB), are also popular as a graft material, having good stability over time and good osteoregenerative properties [8]. However, Chávarri-Prado and coworkers indicated that FDBA during external sinus lift showed an increase in new bone formation compared to DBB [8]. Bone allografts are harvested from human cadavers and subjected to processes that make them viable for transplantation to another person [9]. Allografts can be cortical, cancellous, or corticocancellous, such as freeze-dried bone allograft (FDBA) [10]. Their use was, until recently, avoided due to the absence of osteoinductive potential, the risk of host rejection, and the risk of local infection [9,10]. Also, the high cost and the risk of transmission of viral diseases such as hepatitis B and C and HIV made them less popular at the time [11]. However, the evolution of technology in their sampling as well as the development of gamma ray sterilization have reduced these shortcomings considerably nowadays [9,10,11]. Given their lack of large-scale use until recently, especially in the augmentation of the maxillary sinus floor, as well as different authors’ opinions on their indications and contraindications, we considered that a detailed evaluation of the literature regarding this subject was necessary. 

The aim of this review is to evaluate all scientific articles related to the use of allografts in lateral approach sinus floor augmentation so that their indications are well defined for their future use. 

## 2. Materials and Methods

### 2.1. Participants, Intervention, Comparison, Outcome, Study Type (PICOS) 

This systematic review was written under the PRISMA guidelines [12] and the recommendations of the Cochrane Handbook for Systematic Reviews of Interventions [13]. The focused question was as follows: “In patients with atrophic posterior maxilla (P), what is the efficiency of using allograft bone in lateral sinus lift (I) in comparison with other bone grafts and lateral sinus lift (C) in terms of implant survival rate (ISR); marginal bone loss (MBL), bone volume; new bone formation; biological or prosthetic complications (O)?”.

The PICOS elements were as follows:

- Participants: adults, healthy systemic patients, with atrophic posterior maxilla;
- Intervention: allograft bone and lateral sinus lift;
- Comparison: autogenous, xenograft, or alloplastic bone and lateral sinus lift;
- Outcome: ISR; MBL; bone volume; new bone formation; biological complications; prosthetic complications, radiological or histological assessment;
- Study type: randomized clinical trials (RCTs), prospective controlled clinical trials (CCTs) or prospective, retrospective studies.

### 2.2. Inclusion and Exclusion Criteria for the Included Studies

The inclusion criteria were comparison of allograft alone vs. other types of bone substitutes and sinus floor elevation in the same study. The exclusion criteria were in vitro studies; animal studies; systematic or literature reviews, case reports, case series, monographs, letters to the editor; studies with insufficient, missing, or unpublished data; articles published in languages other than English.

### 2.3. Search Methods

An electronic search was performed by two independent reviewers (A-C.P.-M. and R.L.M.) in the PubMed, Web of Science, and Scopus databases until October 2023. To identify relevant studies, electronic searches were carried out using the following keywords: sinus lift, sinus floor augmentation, sinus floor elevation, sinus membrane elevation, lateral approach sinus floor elevation, osteotome sinus floor elevation, dental implant, titanium implant, standard implant, mineralized bone allograft, fresh-frozen bone allograft, freeze-dried bone allograft, mineralized freeze-dried bone allograft, decalcified freeze-dried bone allograft, autolyzed antigen-extracted allogenic bone, demineralized bone matrix, decellularized extracellular matrix. 

In the first step, titles and abstracts from the electronic searches were screened, and irrelevant articles were excluded. Also, a grey literature search in the OpenGrey and ClinicalTrial databases was performed. A manual search was carried out for the following journals: *Journal of Clinical Periodontology*, *European Journal of Oral Implantology*, *British Journal of Oral and Maxillofacial Surgery*, *Clinical Implant Dentistry and Related Research*, *Clinical Oral Implants Research*, *Clinical Oral Investigations*, *Implant Dentistry*, *International Journal of Oral and Maxillofacial Implants*, *International Journal of Oral and Maxillofacial Surgery*, *International Journal of Periodontics and Restorative Dentistry*, *Journal of Dentistry*, *Journal of Implantology*, *Journal of Maxillofacial and Oral Surgery*, *Journal of Oral and Maxillofacial Surgery and Oral Surgery*, *Oral Medicine*, *Oral Pathology*, and *Oral Radiology*. In the second step, after removing the duplicates, full-text article analysis was performed. Articles that met the inclusion criteria were considered suitable for this review. If any disagreements were present, a third reviewer (A.M.) intervened with a resolution. 

### 2.4. Data Extraction

The following data from the included studies were used: first author, year of study, country, type of study, patients’ characteristics, type of sinus lift surgery, type of bone graft, results, and conclusions.

### 2.5. Risk of Bias 

The Strengthening the Reporting of Observational studies in Epidemiology Statement (STROBE) framework was used for the assessment of risk of bias of the included studies [14].

### 2.6. Statistical Analysis 

Statistical analysis was performed using RevMan (The Cochrane Collaboration 2020, 5.4 version, Oxford, UK) [13]. A random-effects model with a confidence interval (CI) of 95% was used. For the ISR parameter, the risk ratio (RR) (95% CI) was quantified using a chi-squared test (Mantel–Haenszel (M-H)). Due to the heterogeneity detected between studies, a random-effects model was applied in order to analyze effect sizes. For mean bone level (MBL), a weighted mean difference (WMD) (95% CI) with sample size, inverse variance (IV), and standard error was assessed. Statistical significance was considered at *p* < 0.05. The heterogeneity among the included articles was quantified using the I-squared statistic test (*I*^2^); *I*^2^ values were separated into those lower than 30% (low heterogeneity), between 30–60% (moderate heterogeneity), and over 60% (substantial heterogeneity). 

## 3. Results

### 3.1. Study Selection

**Figure 1:**
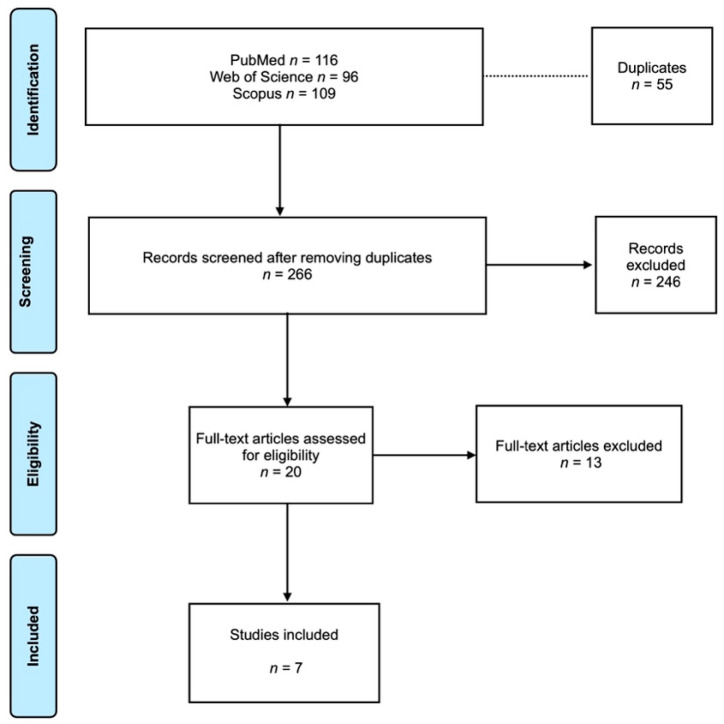
PRISMA flowchart.

A total of 321 articles were retrieved from the selected electronic databases (PubMed, 116; Web of Science, 96; Scopus, 109). After removing the duplicates, a total of 266 articles were screened through title and abstract. In the next step, the full texts of 20 articles were assessed, and 7 articles were included in this review [15,16,17,18,19,20,21]. The flow diagram according to the PRISMA guidelines is presented in Figure 1, and the reasons for article exclusion are shown in Table 1. The coefficient of Cohen’s “K” for inter-reviewer agreement was 0.95. 

### 3.2. Description of the Included Studies

The included articles were published between 2013 and 2023 and were conducted in Belgium, Iran, Israel, Brazil, Turkey, and Italy. The study designs consisted in four prospective and three retrospective studies (Table 2). The total number of patients was 238 (male, *n* = 129; female, *n* = 109). The technique used for the elevation of the sinus membrane was a lateral window with a bone graft with a resorbable collagen membrane. The one grafts used for sinus lift were demineralized freeze-dried bone allograft (DFDBA) (*n* = 67), FDBA (*n* = 76), DBB (*n* = 128), BCP (*n* = 13), and autogenous bone (*n* = 22). Dental implants were inserted during the same surgery as the sinus lift or 6–9 months after the previous surgery. The types of prosthetics used for loading included crowns, fixed partial dentures, or overdentures. Histomorphometry was assessed in two studies [17,20], in which FDBA was compared with BCP or autogenous bone.

### 3.3. Risk of Bias Assessment

The articles included in this review were considered to have a good risk of bias according to the STROBE criteria. The results of the assessment are presented in Table 3.

### 3.4. Statistical Analysis

**Figure 2:**
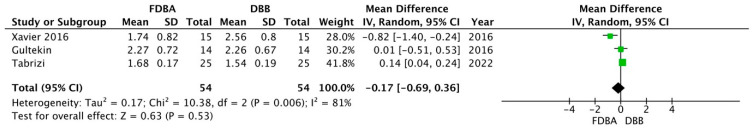
Forest plot showing bone volume for FDBA and DBB 6–9 months after sinus lift [16,18,19].

**Figure 3:**
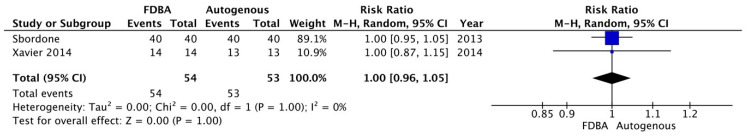
Forest plot showing the implant survival rate for FDBA and autogenous bone 6–12 months after implant placement [20,21].

Statistical analysis could be performed for mean bone volume 6–9 months after sinus lift procedure and implant survival rate after 6–12 months. For bone volume, the comparison was conducted between FDBA and DBB. Mean bone volume indicated a WMD of −0.17 [−0.69, 0.36] (95% CI) with a high grade of heterogeneity (*I*^2^ = 81%); statistical significance was not achieved (*p* = 0.53; Figure 2). For ISR, the comparison was performed between FDBA and autogenous bone. ISR indicated an RR of 1.00 [0.96, 1.05] (95% CI) with low heterogeneity (*I*^2^ = 0%), and the random-effects model was *p* = 1.00 (Figure 3).

## 4. Discussion

Regardless of the method chosen for adding bone to the maxillary sinus, in order to perform a successful implant insertion, a suitable bone supply is required [10,11,12,13]. From a biomechanical point of view, the current literature has shown that a larger surface of the maxillary sinus decreases the mechanical stress around dental implants [35]. In severe atrophies where it is necessary to elevate the sinus membrane for multiple dental implant insertions, it is necessary to approach the sinus floor through a lateral approach [10]. The aim of this systematic review was to assess the efficiency of allograft in sinus floor elevation. The technique of choice for sinus lift (S.L.) in all the included articles was the lateral window approach with collagen membrane and allograft (DFDBA or FDBA) or other types of bone graft (DBB, BCP, autogenous bone). In the meta-analysis conducted by Shah and coworkers, ISR was not influenced by the type of S.L. approach (direct: 0.9691, 95% CI, *p* = 0.688 vs. indirect: 0.970, *p* = 0.686, 95% CI, *p* = 0.686) [35]. These results were obtained by respecting the indications of each technique depending on the anatomy of each individual [35].

In our analysis, ISR was calculated from three studies in which the comparison was made between FDBA and DBB with a limited follow-up of 6–9 months (Figure 2). Another factor that should be taken into account by clinicians is the use of short implants in atrophic posterior maxilla. Our group published a meta-analysis [36] that compared short implants, standard implants, and S.L. It was concluded that after 5 years of follow-up from the included RCTs, standard implants and S.L. showed a higher ISR, even though statistical significance was not obtained (RR 0.97 [0.94, 1.00] (95% CI), *p* = 0.07). Under well-chosen conditions respecting the indications and contraindications for each patient, short implants can represent a sustainable alternative to sinus lift procedures when the bone supply in the molar region allows it [36]. They can be successfully used both in single and multidental fixed prostheses, reducing the rate of complications, operative time, as well as the total costs [36]. Lie San and coworkers conducted a meta-analysis comparing the efficiencies of grafted vs. nongrafted S.L. [37]. The authors concluded that graftless S.L. showed lower height gain and bone density, but the values of implant stability quotient showed no differences between the two techniques [37]. If the surgical technique is respected and the sinus membrane is not perforated, sinus lift without the use of bone grafts reduces the chances of local complications [37]. The blood clot formed between the sinus floor and the Schneiderian membrane ensures a sufficient osteoregenerative potential in well-chosen cases [37]. Under these conditions, the insertion of the implants can be performed without substantial technical problems [37]. However, the residual bone height necessary, the number of implants desired for insertion, as well as the patient’s associated pathologies must be taken into consideration before using graftless sinus lift techniques [37].

In our review, mean bone volume was calculated from two studies that compared FDBA and autogenous bone. The study of Lisa and coworkers [15] compared DFDA and DBB, in which the ISR for DFBA was 81/84 implants and for DBB was 106/107 at an average follow-up of 3.6 years. When FDBA was compared to DBB, at 6 months post-stage-two sinus lift, Xavier and coworkers [18] mentioned one implant failure due to osseointegration in the FDBA group. Gultekin and coworkers [19], in their comparison between FDBA and DBB at 2 years follow-up, indicated no implant failures in either group.

Prosthetic loading was mentioned in three studies (Table 2). Lisa and coworkers [15] mentioned that patients received crowns (*n* = 54; 50.5%), fixed partial dentures (*n* = 24; 22.4%), and overdentures on implants (*n* = 25; 23.4%), with an equal level of satisfaction between patients (*p* = 0.085). The other two studies did not mention what type of final prosthodontic was used. Gultekin and coworkers [19] used cemented porcelain fused to metal crowns or bridges.

In regard to the histological assessment, statistical analyses could not be conducted due to the fact that only two articles compared bone grafts. Xavier and coworkers 2014 [20] compared autogenous bone and allogeneic femoral head. They compared the success of sinus lift with autograft and nonvascularized allograft in 15 patients. They concluded that, overall, their results, regardless of the graft used, were similar [20]. Kolerman and coworkers [17] conducted a study on 13 patients, and they performed a bilateral sinus lift on all patients, comparing the success of the procedure, and indicated that FDBA was superior to BCP. 

The meta-analysis of Al-Moraissi [38] aimed to determine which type of bone graft can produce higher new bone formation (NB) and lower residual graft (RG) and connective tissue (CT) following S.L. After >6 months post-S.L., NB was higher for autogenous bone than for alloplastic bone (WMD −7.06%, 95% CI [−12.59; −1.52]). The bone grafts ranked with the lowest RG were bone morphogenetic proteins, autogenous bone alone, and combinations of autogenous bone and alloplastic bone. Finally, the bone grafts with the lowest CT were the combination of xenogeneic and alloplastic bone, alloplastic bone alone, and mesenchymal stem cells [38]. This study reinforces the idea of the opportunity to combine xenogeneic material with other materials with osteoregenerative properties in order to obtain enough bone height at the floor of the maxillary sinus.

This systematic review had several limitations due to heterogeneity and lack of available information. The main limitation was the low number of the available RCTs. Such studies are absolutely necessary to define these aspects. In the future, we encourage authors to conduct RCTs regarding sinus floor grafting, so that the limitations of our study gradually decrease over time. Secondly, the articles included contained limited or no information about ISR, MBL, biological, or prosthetic complications. Thirdly, histological analysis was also limited. In the analysis of the success of bone grafting, histological studies are a necessity. Carrying them out in the future will reduce the barriers we face today. Furthermore, in the included articles, there was insufficient information about the type of implant, prosthetic loading time, follow-up periods, and complications. 

## 5. Conclusions

The available evidence suggested that allograft bone can be used in external sinus lift. The included articles had a short follow-up time. Future clinical trials with a longer follow-up time are needed in order to indicate the advantages of allograft bone in comparison to xenogeneic, alloplastic, or autogenous bone.

## Figures and Tables

**Table 1:** Reasons for article exclusion.

Author	Reason
Kungvarnchaikul [22]	No data available according to inclusion criteria
Karagah [23]	No data available according to inclusion criteria
Grasso [24]	No comparison with allograft bone
Albanese [25]	No comparison of allograft bone with other bone grafts
La Monaca [26]	Results presented as percentages (%)
Marton [27]	No data available according to inclusion criteria
Galindo-Moreno [28]	Allograft bone alone was not compared
Kolerman [29]	Data comparable with Kolerman [17]
Deluiz [30]	Only allograft bone was used
Sehn [31]	Comparison with composite graft
Ungor [32]	No comparison with allograft bone
Kim [33]	Comparison with composite graft
Viscioni [34]	No data available according to inclusion criteria

**Table 2:** Descriptions of the included studies.

Author. Year. Country.	Type of Study	Patients	Type of Sinus Lift and Bone Graft	Results	Conclusions
Lisa. 2023. Belgium [15]	Multicentric Retrospective	*n* = 107 DFDBA: Mean age: 54.4 ± 12.1 Male: 27 Female: 28 DBB: Mean age: 56.4 ± 11.6 Male: 25 Female: 27	Lateral window, two stage, one stage DFDBA (*n* = 67) DBB (*n* = 74)	Implants 191 implants 84 implants with DFDBA—3 implants failure due to osseointegration 107 implants with DBB—1 implant failure due to osseointegration Mean follow-up—3.4 years Prosthetic loading Crown: DFDBA (*n* = 30); DBB (*n* = 24) Fixed partial denture: DFDBA (*n* = 12); DBB (*n* = 12) Overdenture DFDBA (*n* = 11); DBB (*n* = 12)	DFDBA can be a reliable bone graft for S.L. with a success rate comparable to that of DBB.
Tabrizi. 2022. Iran [16]	Prospective Cohort	*n* = 50 DBB: Mean age: 51.16 ± 9.99 Male: 12 Female: 13 FDBA: Mean age: 51.36 ± 11.89 Male: 15 Female: 10	Lateral window, Stage: NA DBB (*n* = 25) FDBA (*n* = 25)	CBCT Mean bone volume—immediately after S.L. DBB: 1.79 ± 0.16 cm^3^ FDBA: 1.74 ± 0.15 cm^3^ Mean bone volume—after 9 months of S.L. DBB: 1.54 ± 0.19 cm^3^ FDBA: 1.68 ± 0.17 cm^3^ Prosthetic loading—NA	DBB had higher bone density and volume change was less than that of FDBA.
Kolerman. 2019. Israel [17]	Prospective randomized split-mouth	*n* = 13 Mean age: 57.8 ± 6.4 Male: 6 Female: 7	Lateral window, two stages FDBA (*n* = 13) BCP (*n* = 13)	Histomorphometry—after 6 months of S.L. FDBA NB: 21.1 ± 12.8 RG: 28.3 ± 8.2 BCP NB: 14.3 ± 0.6 RG: 34.4 ± 1.3 Prosthetic loading—NA	FDBA was superior to BCP.
Xavier. 2016. Brazil [18]	Prospective	*n* = 30 Mean age: 51.17 ± 10.86 Male: 11 Female: 19	Lateral window, two stages FDBA (*n* = 15) DBB (*n* = 15)	CBCT Mean bone volume—before S.L. FDBA: 2.48 ± 0.72 cm^3^ DBB: 2.9 ± 0.9 cm^3^ Mean bone volume—after 6 months of S.L. FDBA: 1.74 ± 0.82 cm^3^ DBB: 2.56 ± 0.8 cm^3^ Implants FDBA: 35 implants—1 implant failure due to osseointegration—6 months DBB: 35 implants—no implant failure—6 months Prosthetic loading—type of prosthetic NA	DBB offered better results, although both materials can be used in S.L.
Gultekin. 2016. Turkey [19]	Retrospective	*n* = 39 Male: 17 Mean age: 50.23 ± 11.44 Female: 22 Mean age: 53.5 ± 10.62	Lateral window, two stages DBB (*n* = 14) FDBA (*n* = 14) FDBA + DFDBA (*n* = 12)	CBCT Mean bone volume—2 weeks after S.L. DBB: 2.45 ± 0.67 cm^3^ FDBA: 2.82 ± 0.86 cm^3^ FDBA + DFDBA: 2.14 ± 0.59 cm^3^ Mean bone volume—6 months after S.L. DBB: 2.26 ± 0.67 cm^3^ FDBA: 2.27 ± 0.72 cm^3^ FDBA + DFDBA: 1.61 ± 0.44 cm^3^ Implants 77 implants: no failure during 2-year follow-up Prosthetic loading—cemented fixed prosthetic restoration porcelain fused to metal (crown or bridge)	DBB offered greater volume stability than FDBA and DFDBA.
Xavier. 2015 Brazil [20]	Prospective randomized split-mouth	*n* = 15 Mean age: 54 Male: 8 Female: 7	Lateral window, two stages Autogenous bone (*n* = 15) Allogeneic femoral head (*n* = 15)	Histomorphometry Autogenous bone CT: 55.64 ± 14.5 NB: 8.27 ± 3.35 RG: 36.09 ± 12.84 Allogeneic bone CT: 56.81 ± 7.28 NB: 8.26 ± 3.04 RG: 34.93 ± 6.4 Implants Autogenous bone: 40 implants Allogeneic bone: 40 implants Before loading: failure—2 implants After loading: no failure—6 months Prosthetic loading—type of prosthetic NA	The results of autogenous and allogeneic bone were similar.
Sbordone. 2014 Italy [21]	Retrospective	*n* = 14 Mean age: 51.1 ± 8.9 Male: 8 Female: 6	Lateral window, two stages Autogenous iliac crest (*n* = 7) FDBA bone block (*n* = 7)	CBCT 6 months: Autogenous iliac crest: 1.44 ± 0.98 cm^3^ FDBA bone block: 1.94 ± 0.77 cm^3^ 18 months: Autogenous iliac crest: 1.78 ± 0.69 cm^3^ FDBA bone block: 1.44 ± 0.12 cm^3.^ Implants Autogenous iliac crest: 13 implants FDBA bone block: 14 implants At 1 year after loading: no failure Prosthetic loading—type of prosthetic NA	Short-term S.L. using autogenous bone showed similar results to FDBA.

BCP: biphasic calcium phosphate; CT: connective tissue; DFDBA: demineralized freeze-dried bone allograft; DBB: deproteinized bovine bone; FDBA: freeze-dried bone allograft; NA: not available; NB: new bone; S.L.: sinus lift; RG: residual graft particles.

**Table 3:** Risk of bias assessment.

Author	Study Design	Participants	Sample Size	Variable Description	Potential Confounders	Outcome Measurement	Statistical Analysis	ROB
Lisa [15]	1	1	1	1	1	1	1	7
Tabrizi [16]	1	1	1	1	1	1	1	7
Kolerman [17]	1	1	0	1	1	1	1	6
Xavier 2016 [18]	1	1	0	1	1	1	1	6
Gultekin [19]	1	1	1	1	1	1	1	7
Xavier 2014 [20]	1	1	0	1	1	1	1	6
Sbordone [21]	1	1	1	1	1	1	1	7

## Data Availability

Not applicable.
